# Affordance in Socio-Material Networks: A Mixed Method Study of Researchers' Groups and Analog-Digital Objects

**DOI:** 10.3389/fpsyg.2021.672155

**Published:** 2021-08-03

**Authors:** Simone Belli

**Affiliations:** Department of Social Anthropology and Social Psychology, Complutense University of Madrid, Madrid, Spain

**Keywords:** socio-material network, analog and digital tools, researchers, affordance, emotions, socio-material approaches

## Abstract

In this paper, we argue that we can better understand the relationship between social interaction and materiality by linking qualitative analysis of analog and digital practices, adopting Basov's model of socio-material networks. Our research questions turn about the interrogation of how social links distress the usage of analog and digital objects by researchers. We consider scientific networks with the relationship between researchers and their tools as a three-level social material network. It sheds light on how different types of researchers position their engagement with analog and digital materiality over time and its affordance and emotional attachment. This study contributes to the understanding of researchers' practices that involve new and old techniques and specific and not-specific tools.

## Introduction

Scientific collaboration is defined as “the working together of researchers to achieve the common goal of producing new scientific knowledge” (Katz and Martin, 1997). We will adopt Basov's model (Basov, 2018) of socio-material network analysis to focus on how researchers from different disciplines and institutions use analog and digital tools to collaborate between them. We identify the relations between analog and digital tools and its affordance, just like interpersonal links constitute the texture of the social.

**Figure 1:**
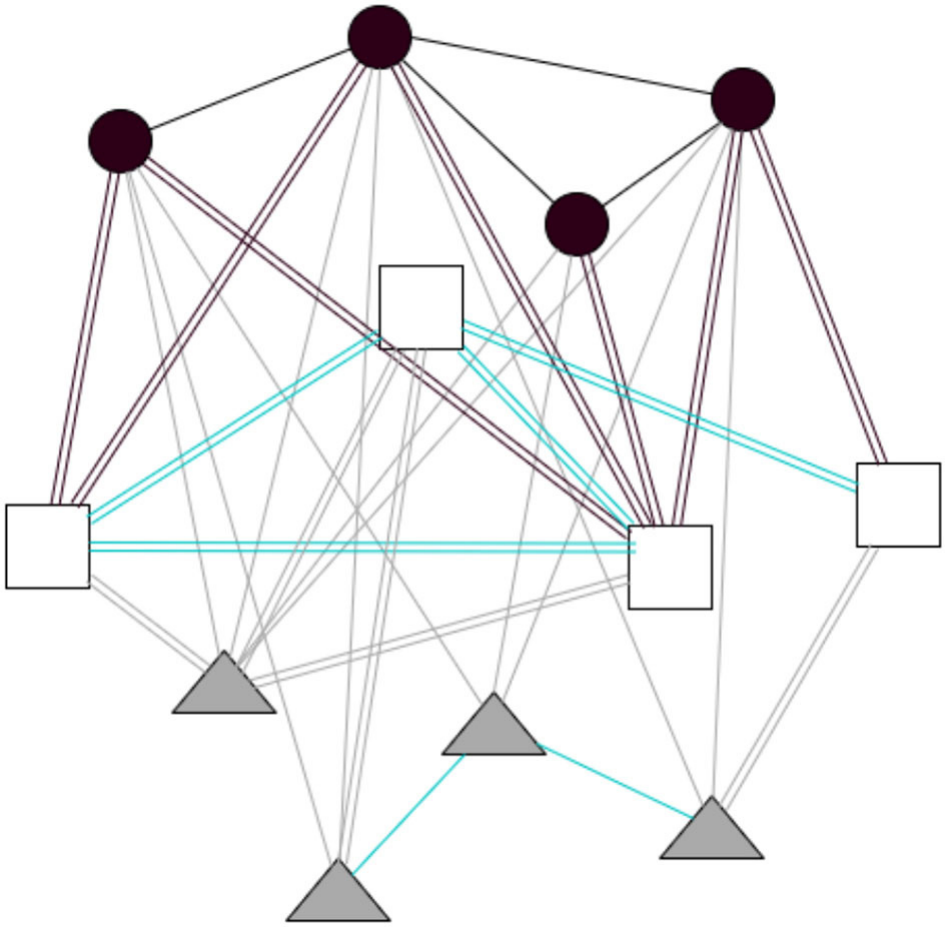
A socio-material network based on Basov (2018). Circles, actors; Triangles, analog objects; Squares, digital objects; Black lines, social ties; Cyan lines, material links; Gray lines, usage of analog objects; Black double lines, usage of digital objects; Cyan double lines, digital links; Gray double lines, analog/digital links.

Basov (2018) argues that network analysis usually observes specific structures of social links. In his study, he presents a model to capture the engagement between individuals from different collectives and materiality. In this paper, we introduce a third type of relations, causing the digital transformation of this materiality. This three-mode network of objects' usage connects participants to analog and digital items, capturing the connection between these three orders characterized by two one-mode networks: social ties and links between analog and digital objects. Then, we can examine “socio-material networks” (see Figure 1) that contain the three types of relations: (1) social links, (2) relations between items as they are shared and collocated in the analog and digital space, and (3) relations between actors and items they use in their practices (Basov, 2018) and affordances.

Such an extension permits a representation of network designs presenting how socially linked actors use analog and digital items and, in this way, together involve with material structure. For example, when two researchers use specific tools to accomplish their work, we can view it as a two-level cycle where nodes are two objects and two actors. Two boundaries are item usages: one edge is a teamwork link; and the other edge represents a link between instruments (Basov, 2018), when an analog object links with a digital object, for example, the hardware and the software, or the printed book and its digital version. We will introduce an analysis to observe a more connected world between objects. The internet of things (IoT) is one of the aspects where actors often do not enter in this relation, and it can be explained with the gray double lines of Figure 1.

This document develops a qualitative technique approach based on Basov's model (Basov, 2018). It starts by gathering a set of ethnographical data, such as interviews and notes. This is used to construct the three-level networks. After that, modeling outcomes are contextualized using ethnographic information. Zooming in on specific settings using ethnography, the physical and the social turn out to be intertwined in ways that lead academics to accept ethnographic studies to speak about “sociomaterial practices” (van Dijk and Rietveld, 2017). The mixed method applied in this paper is planned to be perceptive to the setting, discovering the configurations beyond those caught by an ethnographer's perceptiveness (De Nooy, 2009). We chose Basov's model for this study because it identifies the strong relationship between the materiality in interactive and professional relations. This model has been widely justified and empirically tested by different traditions of qualitative methods. Bridging methods with this model have been the main source of theoretical and methodological innovations. In social network analysis the main focus has traditionally been on positions of actors and their connections. The latter served to describe actors' characteristics, while other types of relations were linked, i.e., how social ties affect engagement of individuals with similar materiality in a shared space over time (White et al., 1976; Wellman and Berkowitz, 1988; Wasserman and Faust, 1994; Borgatti and Foster, 2003; Basov, 2018). Our research questions revolve around the interrogation of how social ties affect the usage of analog and digital objects of researchers with similar backgrounds and experiences over time. We started with two research questions for designing the investigation:

Q1. What is the affordance of analog and digital objects used by researchers in their activities?
Q2. What is the role of digital technology in the socio-material networks and how do these affect researchers?

This research contributes to the understanding of academics' practices that involve new and old methods and specific and not-specific items. The study was planned and applied to academics within the framework of the EULAC-Focus project. The investigators asked use different disciplines and are located in different EU-CELAC countries. The main purpose is to analyze how information and communication tools have affected the mode researchers are related. Moreover, we have collected ethnographic data observing how researchers engaged in their scientific collaborations, exchange data, informally interact, and hung out with colleagues, witnessing how they use analog and digital items in these social interaction practices. This allowed an inquiry into how everyday and work-related interactions structure material contexts (Basov, 2018) and its affordance.

## Affordance in Socio-Material Structuring

This paper explores the socio-material networks of the work practices and the affordance of analog and digital tools. Investigation in technology focuses on the effect of communication, materiality, and emotion, but affordance may be one way to synthesize these.

Socio-material approaches attempt to understand the constitutive entanglement of the social and the material in professionals' activities (Orlikowski, 2007). This provides insights on the importance of personal communication and social interaction processes for the cluster and shows the high relevance of network analysis for studying this case (Basov and Minina, 2018). In short, the question is whether individuals choose their objects because of the similarity of their characteristics, in other words, a sort of contagion (Basov, 2018). Yet, concerning material objects, it seems to be interesting to test for a hybrid principle (Mark, 1998), i.e., the impact of homophilous ties (ties between individuals with similar stable characteristics, such as gender and age) on the achievement of similar material preferences (Basov, 2018). For example, we can expect two researchers of the same gender, who hold a similar professional position, and who work in the same area of research, to use similar sets of analog and digital tools in their work.

Nowadays, we comprehend analog and digital objects as specific tools used by researchers for their functionality and the broader (especially the digital) research infrastructure designed to support researchers' work (De la Flor et al., 2010; Blanke and Hedges, 2013). This type of environment is where researchers survey everyday work. Many of them work on international projects with colleagues from different institutions of the same country (53% of the researchers surveyed) or from other countries (38%). They experiment in their activity with what is considered the digital transformation of science. Digital infrastructure is designed for large projects to enable collaboration (Simeone et al., 2011). However, in these works that we have mentioned, authors have not explored the impact of digital technologies—that is, new tools being developed, such as text analysis tools, and everyday digital resources, such as Google Docs and Skype—within the context of scholars' views of their daily research practices (Given and Willson, 2018).

When people interact through technologies, they must discover modes of handling the limitations on the opportunities for action that occur from those technologies' affordances (Allen, 2013). Gibson (1979), who coined the term of affordance, argues that by allowing the different affordances that constrain both the possible meanings and the potential uses, we can analyze precisely what the effects and constraints associated with technological forms are. Socio-material networks do not take the individual or the group as a unit of analysis, but focus on the practices, on how these practices are relationally composed and enact particular sorts of actors (Law, 2009; Gherardi, 2012; Decuypere, 2019). Actor-networks are instable structures of associations performed into existence by the actors involved and involve humans and non-humans. The stability of a network needs the continual “translation” of interests, which between humans is equivalent to negotiating the arrangement of aims and concerns (Latour, 1991).

Parchoma (2014) introduced the concept of “technological affordance,” examining the interactions between human societies and their technologies. Examining social and political effects of technological innovations, academics have assumed growth to be a technological determinist viewpoint that holds that new technologies “actively caus[ing] new forms of social relations to come about” (Hutchby, 2001, p. 442). Subject–object and direction of agency discussions reinforce challenging discourses on interactions between objects and human practices. Therefore, Hutchby explores, reduced possible relations between human actors and technological objects offer a route out of the subject–object and direction of agency impasse.

Norman's (1999) introduction of perceived affordances goes some way toward resolving how, as well as what, an object can afford an actor via an opportunity to select it and to interact with it. The difference between real (physical) and perceived (cognitive) affordances remains tricky. Norman's real technological affordances persist in the realm of the creator, beyond users' capability to recognize, choose, or act on them, and therefore, beyond academics' capacities to study them (Parchoma, 2014). Oliver (2005, p. 406) resists that there is an ontological impasse in Norman's conceptualization of perceived affordances because “all we have access to is what we can perceive—thus all we can ever access are ‘perceived' affordances.” Consequently, Oliver debates that Norman fails to address the ontological discrepancy of at once holding an interpretive view and constructing the positivistic claim that affordances are objective properties of the world (Parchoma, 2014). Affordance has been provided between technological determinism and social construction, a change that made it possible to point to the materiality or purposes of technology by remembering that these tasks are permanently included in the activities of users (Graves, 2007; Neff et al., 2012; Nagy and Neff, 2015).

## Data and Method

### Empirical Setting and Data Collection

This research focuses on the socio-material networks that occur in research groups in their daily practices. For this reason, we have decided to develop a qualitative fieldwork combining discursive production through semi-structured interviews with ethnographic observation practices. We have also related researcher's stories and speeches to the records of practices during work time, exploring the socio-material organization of teamwork and the meanings and affects that implies. We consider this approach the most appropriate to look into the nature of actors (humans and/or tools), their affordance, the cognitive implications of their interactions, the phases, the states of the productive trajectories, and the new results. The selection of cross-discipline case studies produce data on the existence of both commonalities and discipline differences depending on the scientific contexts and their institutional environments.

**Figure 2:**
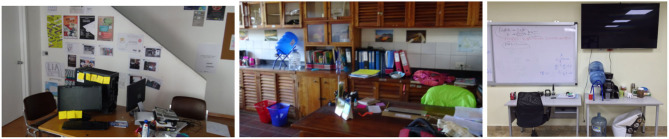
Objects in academic spaces (offices, laboratories, and field works).

In each case we discovered spaces where researchers work. These are filled with thousands of analog objects that, in many cases, are linked to digital objects. As it was expected, these objects were both work-related, such as books, tools, equipment, and scientific materials, and daily items, like household objects, furniture, dishes, food, clothes, and consumer electronics (Basov, 2018; see Figure 2 for some illustrations).

**Figure 3:**
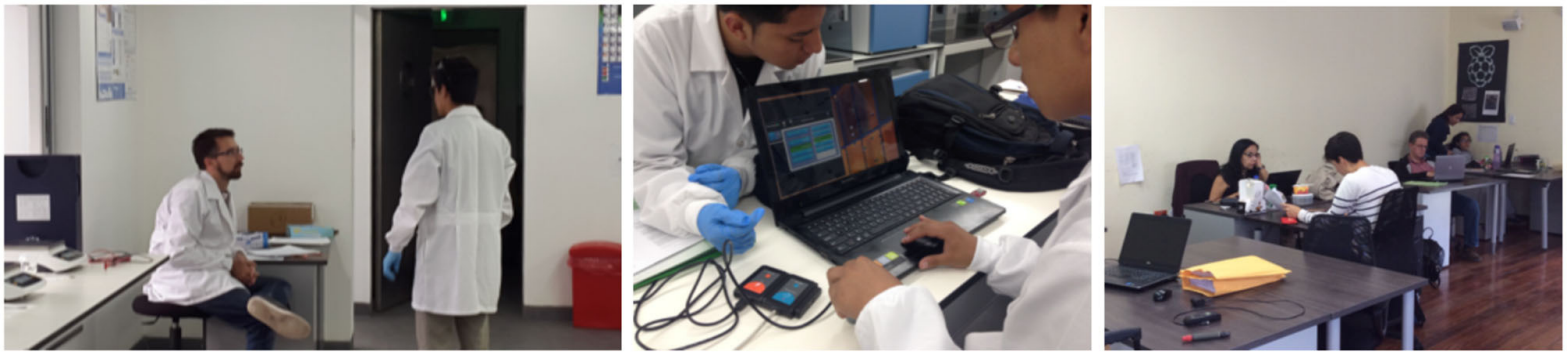
Different types of interactions in academic spaces (offices, laboratories, and field works).

During data collection, we observed how researchers engaged in discussions and joint projects, exchanged information, casually interacted, and hung out with colleagues, witnessing how they utilize analog and digital objects in these social processes (see Figure 3 for illustrations). This enabled an inquiry into how every day and work-related interactions structure material contexts (Basov, 2018) and its affordance.

The data we analyze in this paper was collected in three waves with an 8-month gap between them. The initial wave began in November 2015, the subsequent wave in July 2016, and the third wave in March 2017. The analysis of these data finished in February 2020. In each wave, the data was gathered following the actions defined below. Each wave took 2 months. These were followed by semi-structured interviews (a total of 125 in the three waves) with each of the 51 researchers from one to three interviews for each depending on the availability of the scientific. In each wave, each of the studies began with an excursion around the space, providing data on the preparation of the material scenery. Researchers were selected from a list of the most productive researchers in their universities, with more than 20 contributions in Web of Science and Scopus. Participants were asked about the types of research in which they are engaged to contextualize discussions of specific technology needs and research practices. The main characteristics of researchers are summarized in Table 1.

**Table 1:** Main characteristics of researchers.

	**Anonymized name**	**Country**	**Gender**	**Age**	**Area**
1	Karl	Portugal	Male	37	Anthropology
2	Marc	Peru	Male	39	Architecture
3	Cristina	Bulgaria	Female	41	–
4	Otto	Cuba	Male	48	Biology
5	Fran	Spain	Male	49	–
6	Veronica	Venezuela	Female	62	–
7	Fred	Germany	Male	52	–
8	Hector	Ecuador	Male	44	–
9	Gina	Argentina	Female	41	–
10	Viviane	France	Female	34	–
11	Helena	Cuba	Female	46	Chemistry
12	Flavia	Spain	Female	33	–
13	Gil	Ecuador	Male	45	Communication
14	Gail	Ecuador	Female	42	–
15	Martin	Spain	Male	56	–
16	Iris	Ecuador	Female	40	–
17	Eduardo	Spain	Male	34	Computation sciences
18	David	Ecuador	Male	43	–
19	Vincent	Spain	Male	37	–
20	Jorge	Spain	Male	39	–
21	Rafael	Ecuador	Male	31	Design engineering
22	Rossy	Spain	Female	45	Education
23	Jane	Brazil	Female	49	Environmental sciences
24	Lian	United States	Male	37	Geology
25	Alberto	Mexico	Male	35	–
26	Ivan	Mexico	Male	34	–
27	Erick	Colombia	Male	37	–
28	Phoebe	Netherland	Female	45	–
29	Monica	Costa Rica	Female	56	Health sciences
30	Ryan	Austria	Male	57	–
31	Gaston	Mexico	Male	38	Humanities
32	Javier	Ecuador	Male	37	–
33	Ian	Bolivia	Male	35	–
34	Wanda	Spain	Female	32	–
35	Ismael	Spain	Male	54	Information sciences
36	Nina	Belgium	Female	61	Law
37	Jeac	France	Male	45	Math
38	Juan	Mexico	Male	42	–
39	Carl	Uruguay	Male	31	–
40	Mary	Greece	Female	47	–
41	Roland	Uruguay	Male	45	Odontology
42	Harry	Ecuador	Male	42	Physics
43	Gabriela	Argentina	Female	41	–
44	Jose	Spain	Male	37	–
45	Axel	Colombia	Male	43	–
46	Luciano	Chile	Male	46	–
47	Diego	Argentina	Male	56	Political sciences
48	Yvonne	Brazil	Female	48	–
49	Kelly	United Kingdom	Female	36	Psychology
50	Pino	Italy	Male	42	Sociology
51	Patrizio	Italy	Male	41	–

We also conducted photo elicitations (168) with every member, successively presenting about 20 photos of the common zones filled with items to encourage reporting about them and their usage, and recorded 139 h of video interactions between researchers and objects. The observation of the process was completed with systematic interviews with the participants in the work process. Moreover, the objective account of behavior cannot be completed without the subjective insight of all the actors in the research process. Since our model is both integrated and cross-disciplinary, we want all the teams to work together from minute 0. Fieldwork calls for the implementation of long periods of observation of the actual Innovation Process, with HD cameras and note-taking.

Each researcher defined their unit of analysis, taking into account the theoretical and methodological part, which explains expertise, skill, and the acquisition of competences as a socially defined element of the work process. Moreover, the data available, both the notes and video archives, were codified openly following a thematic axis that looks at the work process globally, as a unit, as well as the micro-interactions that have a sense in themselves and that are part of the whole. To codify words and images together, we used the software Atlas.ti version 8.

The fieldwork data, both field notes and video recordings, were organized in field diaries that describe the work process in its external dimensions (space and time contexts), indicating participants, actors, and technological instruments that are part of it as well.

This initial organization, with the application of an Excel template of coding, facilitated intercoder reliability and the common analysis of data. As a methodological innovation, the video data was also organized, with the aid of a professional editor, following two complementary objectives: (1) To expand the information included in the verbal narrative through the synchronization and the accurate time-stamping of the video archive; and (2) To illustrate, complement, and maybe contrast the description that comes from the verbal note-taking through the selection of those images that constitute a whole innovative process.

This theoretical-methodological approach has never been applied to the observation of the innovation in its local environment as part of a distributed system (Hollan et al., 2000), both multimodal and embodied (Alač, 2005; Clark, 2008). Most tools that intervene in the work process possess an internal cognition or a “how-to” affordance that is made manifest only in the “doing” in the moment of interaction. Moreover, studying the work process in the actum, while they are happening through direct and video-aided observation is a unique way of capturing data in the making. The white rooms of experimentation, which constitute the dominant paradigm in social sciences and specifically in cognitive science, cannot explain what happens when people innovate and create in their everyday settings. In all, this comparative case study and its joint in-depth analysis of parallel cognitive ethnographies constitute a first-time opportunity for producing new knowledge about the impact of technological transformations, modifications, and substitutions on the patterns of communication and coordination in teamwork.

We identified the most common terms used by researchers (Table 2). We focused on the 10 used most frequently. The most frequent is “research,” used 927 times in total. We also have “technology,” used 545 times in total. These are the concepts that do not suggest any position-specific research area, but instead refer to the central notions for the whole field of scientific production, such as research, information, university, and so on. However, let us have a look at the quotes made by researchers in the next sections to understand how concepts are used. Several hundred quotes include these shared concepts. Here we provide only a few typical quotes as examples, but many others reveal the same.

**Table 2:** Ten most frequent terms shared by researchers.

**Position**	**Concept**	**Used times**	**rf**
1	Research	927	0.1325
2	Information	894	0.1278
3	Access	799	0.1142
4	University	696	0.0995
5	Project	682	0.0975
6	Digital	681	0.0974
7	Communication	617	0.0882
8	Data	604	0.0863
9	Laboratory	550	0.0786
10	Technology	545	0.0779

The use of these terms in the data collected are often accompanied by terms that relate to objects, which make up the socio-material networks. We have identified 12 objects that researchers used in their daily activity (Table 3). We understand that these objects are very different. Some objects contain information, some are useful for accessing information, and others are digital platforms. Still, these are the most common tools that any researcher uses in their activities. On average, the material network of each researcher consists of nine different analog and digital objects, the most significant being 22. Material network degree centralization of these 12 objects is moderate, so no objects are engaged in significantly more material contexts than the rest.

**Table 3:** Twelve most frequent objects shared by researchers.

**Position**	**Objects**	**Analog or digital**	**Used times**	**rf**
1	Journal	Both	478	0.2324
2	Article	Both	468	0.2275
3	Software	Digital	337	0.1638
4	Computer	Analog	185	0.0899
5	Email	Digital	103	0.0501
6	Google	Digital	95	0.0462
7	Book	Both	91	0.0442
8	Facebook	Digital	89	0.0433
9	Skype	Digital	79	0.0384
10	Scopus	Digital	61	0.0297
11	Whatsapp	Digital	46	0.0224
12	Dropbox	Digital	25	0.0122

Digital objects and practices generated by their use play a significant role in our interviews. We can observe that the first complete analog object that appears in Table 4 is the computer. The computer is the best tool to link researchers to digital objects, as we will observe. In the next sections, the quotes show that even the most common concepts common for researchers are used by them to express different activities related to these objects. However, among the most frequently used concepts in Table 2, it will be important to consider the most frequent specific objects used in the activities of researchers (Table 3). In the first two positions and in position seventh of Table 3, we have three objects that can be analog and digital. Few studies, to date, explore how scholars integrate analog and digital objects into their daily research practices.

**Table 4:** Patterns in socio-material networks based on Basov (2018).

**Illustration**	**Interpretation**
Circle	Actors
Triangle	Analog objects
Squares	Digital objects
Black lines	Social ties
Gray lines	Usage of analog objects
Black double lines	Usage of digital objects
Cyan double lines	Digital links
Gray double lines	Analog/digital links
Red double lines	Not positive emotional attachment of usage of digital objects
Green double lines	Positive emotional attachment of usage of digital objects

### Construction of Socio-Material Networks

Several matrices (27) have been elaborated to include the different types of data collected. Matrices related to relations among items as well as between researchers and items were shaped for each wave individually using the analogous ethnographic information. When an item usage by one of the researchers was directly observed or when an informant mentioned that he or she used an item, a link between the researchers and the item was listed. Each relation was supplemented with data on regularity, duration, and way of usage, when available (Basov, 2018). Only items used by at least two researchers were incorporated in the dataset. The link between elements that show their connection in similar actions were constructed on functional links between objects (if they are used together, for example, a bottle and a glass) or on their material closeness (continuous juxtaposition, for example, a laptop near a microscope) (Riggins, 1990; Basov, 2018).

These results were triangulated with the results of the ethnographic notes and answers about links with other researchers. Information processing resulted in 153 three-level socio-material networks (three networks equivalent to the three waves for each of the 51 researchers) that contained (1) social links of teamwork, (2) ties between items, and (3) item usage. To estimate emotional attachment as predictor of the item usage in networks we use models that includes emotional patterns that combine several types of emotions at the same time. They involve the actors' attributes of objects, their usage, and the relations in collaboration through these objects. Emotional attachments refer to the emotional closeness of the researchers to the objects used. Because we did not want to differentiate between but instead to compare these various interpretations within the two aspects of emotional attachment (positive and not positive), we did not specify any particular emotion. Table 4 describes the patterns included in the network models elaborated with the software Diagrams.

Gender, age, and area of research are stable features, obtained long before we documented usage of items and physical structures in the material spaces of the researchers. The node sets are conformed across the three waves so that only researchers and links present in both waves and wave-specific relations between them are subjected to the analysis. Additionally, we combined networks of all researchers to capture socio-material shaping principles invariant. It allows for testing the relative importance of different principles of socio-material structuring outlined above as they potentially reinforce or compete with each other while accounting for various structuring processes within the social, material, and object usage networks themselves (Basov, 2018).

## Results

### The Affordance of Analog and Digital Objects

With these premises, we observe how technologies are often considered as artifacts that enable researchers to extend their activities in laboratories and offices. Through social interaction in digital environments, researchers can be present in and navigate through multiple places (Nagy and Neff, 2015). We observed how researchers tend to perceive digital tools as invisible entities that are mediated by the human sensory, cognitive, and affective processes (Renò, 2005).

We start this section with the ethnographical observation realized in Harry's workplace (Observation 1).

*Observation 1*

*The physicist (he is a theoretical physicist) connects the laptop and opens his email account to receive the latest data from his experimental colleagues, Carl and Owen, from universities in other countries. After ten minutes (10:10), he reviews these results, and then he opens an Excel file to change some values (10:15). At 10:30, he makes a call via Skype to Carl to request clarification about the project. The call lasts 4 minutes, and then he makes new changes to the previous document. At 10:50, he checks the further information that he has updated, and at 11 am, Harry resends the report to Carl and Owen via email, explaining the change that he has introduced. At 12:20, Harry starts research on the next phase of the project, doing rough calculations until the end of our ethnographical observation, with MatLab software*.

**Figure 4:**
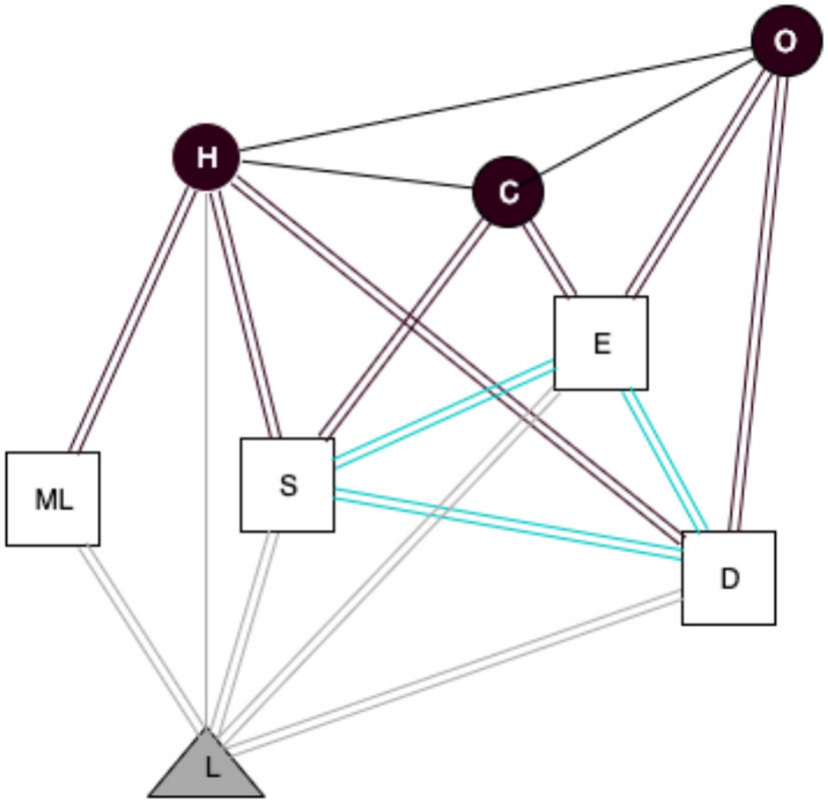
Socio-material network based on Harry's observation. Circle, actors (H, Harry; C, Carl; O, Owen); Triangle, analog objects (L, Laptop); Squares, digital objects (S, Skype; E, Email; D, Document; ML, MatLab); Black lines, social ties; Gray lines, usage of analog objects; Black double lines, usage of digital objects; Cyan double lines, digital links; Gray double lines, analog/digital links.

In Figure 4, we can observe two triadic clusters connected between them. The first one is composed of the three actors (H, C, and O), and the second one is composed of the three digital objects (S, E, and D). The only analog object that appears in this observation is the laptop. The three digital objects used by the researchers are connected between them, but there are not engaged with the same materiality (L), an analog object. We have, in these observations, researchers from the same area of knowledge, Physics, and with the same position and age that use the same digital objects. Of course, the only gray line is the material connection between the main actor, Harry, and the analog object. The affordance of this analog object gives access to several digital objects, three of them connected with the first triadic clusters. Still, we do not consider this connection a material connection because there is not a usage of the analog objects for C and O in the observation. We can hypothesize that these two experimental physicists are connecting from their laptops.

Now, we compare this observation with another one from a researcher of the same area and the same position, Jeac (Observation 2).

*Observation 2*

*Jeac arrives at his office at 9:45 am, and he passes the first ten minutes preparing different documents that contain different information. At 9:55, he started to develop different mathematical models in his analog notebook until 10:30. Then, he opens his laptop and searches an old email from a colleague from another institution, Pietro, in his folder. He finds it and adds the information contained in it in his notebook, then he modifies his calculation and reviews the entire operation (10:50). At 11 am he runs the software C*++ *to add this information*.

**Figure 5:**
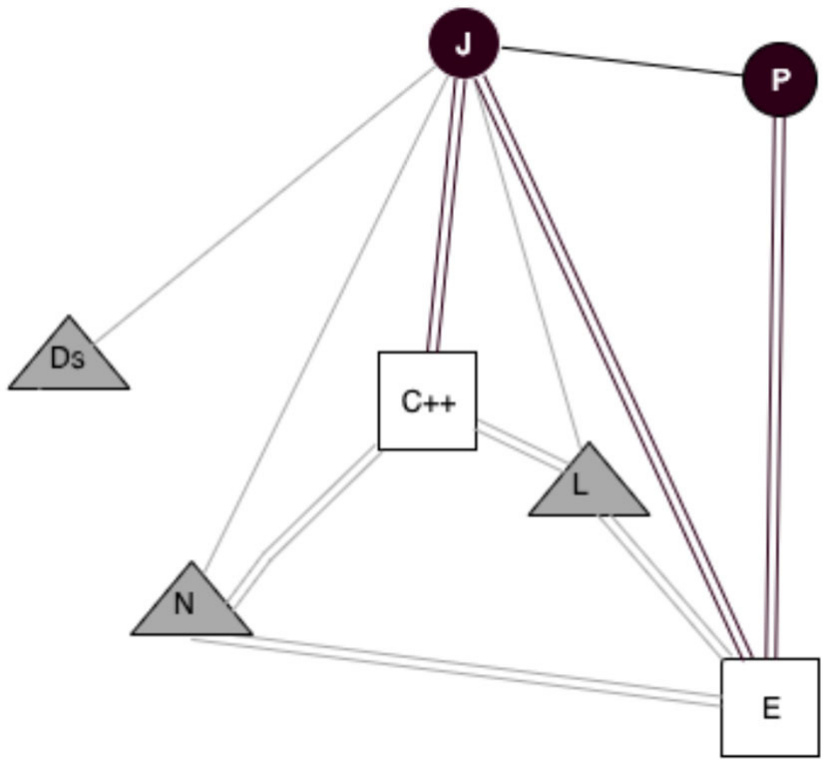
Socio-material network on Jeac's observation. Circle, actors (J, Jeac; P, Pietro); Triangle, analog objects (L, Laptop; Ds, Documents; N, Notebook); Squares, digital objects (C++, C++ Software; E, Email); Black lines, social ties; Gray lines, usage of analog objects; Black double lines, usage of digital objects; Gray double lines, analog/digital links.

In Figure 5, we can observe how the information searched by Jeac in an old email is passed to an analog notebook and not directly to the software C++. In this case, there is not a direct connection between the two digital objects (Email and software). Still, an analog object acts as an intermediary to pass the information. Objects' qualities and features of the objects that we have observed are more focused on research (C++, Python, etc.). Still, others demonstrate the usefulness of not-specific objects (Word, Excel) in scholars' practice.

We can observe how researchers from the same area use the analog and digital objects differently, depending on the users' perception of the affordance of the object used. It appears evident that more researchers are connected in this network; more connections will be linked between digital objects and actors. It is interesting how the triadic structure that we have observed in the previous network (Figure 4) changes in this last network (Figure 5).

The triadic structure is our analysis unit to identify the three levels of analysis between actors, analog objects, and digital objects, that can be resumed and adapted for each context with this statement: Actors, who come from different institutions and countries, use analog and digital objects to share and to communicate information. But as we have observed in the last two networks (Figures 4, 5), it is hard to identify the accurate structure of these triadic clusters. On the other hand, we have seen that when a researcher works individually they tend to work more with analog objects; many of them do not directly connect with digital objects, like analog documents and notebooks. Often, these ideas and information generated in individual researchers pass from the analog objects to digital ones, like the examples of the operations of Harry and Jeac.

Next, we present a large extract from the interview with Patrizio, a 41-year-old from the area of Sociology that allows us to understand in which way the affordance of digital objects is perceived from researchers in their daily activities through the emotional attachment to the objects' qualities and features (Extract 1). In the extract, the researcher explains a typical routine in his daily activity.

*Extract 1*

*Patrizio: My typical routine starts with the computer, where I work mainly with Word, the Internet browser, and different kinds of communication systems like Skype, which is now increasingly our favorite tool to communicate. Then, I brows various types of databases, and I use a lot of Excel, a lot of PowerPoint presentations. Then, I use several different platforms, from Google Scholar to Academia, which I did not consider before, but it is a quite impacting platform on the way scientific knowledge circulates. (…) I have a very busy ResearchGate profile built for curiosity if someone asked for my profile, but it is not updated. And I have an Academia profile where I started to take care of it in a better way but is now not very updated. I think it is an excellent way to circulate your work, but at the same time, for me, it is like this other kind of exploitation of your work, which, if I can do without, I would be much happier*.

*Interviewer: But, you can or not?*

*P: I've not this huge pressure. In the sense that, now I have a profile there and a lot of things I did that I probably will update it when I have time*.

**Figure 6:**
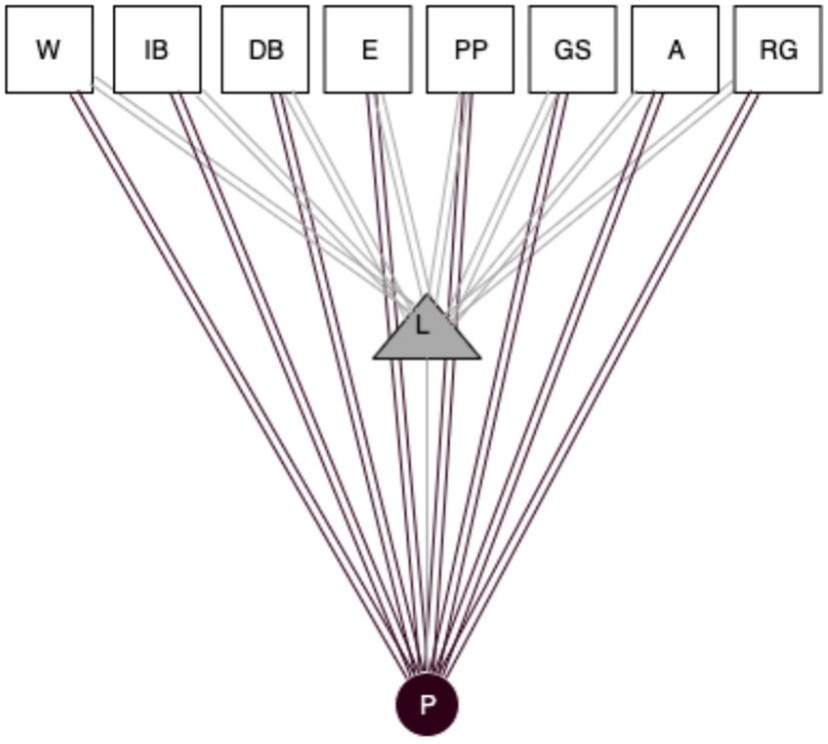
Socio-material network of Patrizio. Circle, actors (P, Patrizio); Triangle, analog objects (C, Computer); Squares, digital objects (W, Word; IB, Internet Browser; DB, Databases; E, Excel; PP, PowerPoint; GS, Google Scholar; A, Academia; RG, ResearchGate); Gray lines, usage of analog objects; Black double lines, usage of digital objects; Gray double lines, analog/digital links.

Patrizio is drawing his triadic network, starting with access through his computer to several digital objects (Figure 6). Each of these objects have several characteristics and gives him several qualities and features that many times are perceived with not positive attitudes, such as updating the profile in these networks. Users' perception of affordances is not always positive, as we can observe in this extract. Researchers do not respond with the same attitudes to the affordances of these digital objects. We follow with another extract of the same researcher to go more deeply in the affordance meaning of these digital objects (Extract 2).

*Extract 2*

*Patrizio: No, I don't use social networks because I'm not very comfortable with their uses. I don't use Facebook; I don't use any social network. I think probably, yes, I don't have, I don't use any kind of social network profile, or Instagram, so I'm not that active with Academia, even if I think that it's important to have a kind of profile on Academia. Because in some way, people look for you in Academia, a good profile, and if I have a good profile with 30 or 40 contents. There would be my profile with a photo, and if I published a call for paper, I posted it there. Well, I also have a website, I have a professional personal website that I care a bit more about partly because I started editing in 2001, so it is quite old and is an excellent tool and some kind of continuity. Also, because it is a right spot for your work well organized in the way you prefer, it could be arranged. And, because yes, so I'm more active with my personal website than with Academia. But, I think that Academia is very important, having a profile in Academia in some sort, and I have one. I just say that I don't like to spend a lot of time on my work on doing this because I think it is in some way also a sort of…. I do not see, I sometimes see people that ask for comments to the paper and the open discussion. But I think this is in the huge complexity stratification and is work that a researcher can do to respond to one question or about something general in the academia. It's quite unusual. In general, then probably there some specific kind probably it's a… I think one time several years ago I contested to a forum on something about the.. I think that could be something that could potentially be very interesting, and if bury something to be a tool interesting and requires a considerable effort which is not for to doing things in the platform, but using this platform to activate, consolidate, and develop different kind of relationships where you want to invest other aspects of your work*.

**Figure 7:**
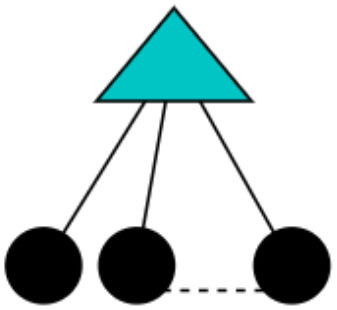
Usage degree of objects of Basov (2018). Black circles, actors; Cyan triangles, objects; Gray lines, usages of objects.

We observe in this extract how digital objects offer a great variety of affordances that analog objects rarely provide too. One of the most important is the connections that a digital object provides to its user, in this case, the other researchers. Using a social network can enable sharing of your research and connecting with researchers from different parts of the world, although we commented before that this affordance gives both positives and not-positives emotions, like Patrizio explains. On the other hand, Patrizio understands that the tool is powerful because it can put an individual in contact with an entire community that reads and comments on the article that they share in the network. This passage moves the protagonist from the actor of the socio-material network to the digital object. It is the article deposited in the open database that connects different researchers and, in consequence, different computers. It is what Basov (2018) called the usage degree of objects that connect different actors (Figure 7).

Of course, this usage degree of objects is not the same for every actor, as we perceived from Patrizio's narrative. This is for the emotional attachment that objects cause to the actors (Umphress et al., 2003; Fang et al., 2015; Basov, 2018). In this case, there is an emotional discordance related to the affordance that the social networks, in this case, Academia.edu network, offer. The materiality of the digital object engages with positive or not-positive attitudes by the actors, and that can stimulate professional activities. A similar emotional attachment tends to be found in homophily networks, with researchers from the same area, age, position, and gender. For example, two researchers from the field of Sociology share the same emotional attachment to these digital objects, the same not positive aptitudes for social networks. At the same time, researchers of the same age also tend to have a similar emotional attachment to similar digital objects.

*Extract 3*

*Patrizio: A digital profile for a scientist is important. In this sense, if you want to get a job, when they want to invite you as speaker somewhere, when someone finds an article from you, and they want to know who you are, who is the author, they look to your profile. So, it is better to have a profile that, in some way, reflects what you do*.

*I: When did all this transformation start?*

*P: When? That is an interesting question. When I began my Ph. D. in 2003–2004, this was not still something new, if there was something, an increased impression of institutional websites that have to be with the correct information. And then, of course, the social networks that grew before outside of Academia was significantly affecting in my vision the way a new generation of scientists started to interact with scientific platforms like Academia. In that sense, it was quite contemporary. Still, it was quite contemporary in the… this is, I think this is a case, we are, generally, we are used to seeing that before digital tools started to be adapted for communication, they began to be adapted for scientific work. And then, the filter they exported, they became also accessible outside of academia. This is true for the internet, for email, or that kind of tool*.

*I: Ok. But in practice, you only mentioned Word, but there are also tools like Evernote, tools for collaborative working, for sharing an article, or*…

*P: I don't like the tools that many people use, for example, Google Docs. I used it several times, and I don't feel very comfortable with it*.

*I: Why not comfortable with it?*

*P: I'm not comfortable because I don't know how to use it. Word is more performative in several different senses. Different people working at the same time in Google Docs with the same document, and this is very powerful. But, at the same time, there is an alternative. The alternative, of course, is using materials on Dropbox. And, in Dropbox you can use Word, and at the same time, you have a register to sharing, and how can I say? A sort of possibility to have a contemporary process that occurs*.

*I: For example, a typical situation where you and the colleagues share documents or produce documents together by Dropbox?*

*P: By the way, I use Dropbox for all the stuff that I do*.

*I: In what, you use it?*

*P: For all the stuff I work on except things that I only work by myself. I have a Dropbox folder for any project that I have activated with other people, which could be up to ten folders. This is useful in terms of backup. In the sense that, if something is there also if you lose your computer, and because it is there, you are not just the only person with the responsibility of keeping files. All people can get the last version of the data. The name is when there is something in the right at the top of the screen where there say that your friend or your colleague is working at that moment. This is very useful, and this is clearly. I think it is radically changing the collaborative process of working together and is not something new with Dropbox, it is something that came from the way enterprise, internet, systems. That started to be shared in enterprises since they have this kind of possibility. But this is Dropbox, of course, today it is very popular, or not just popular, but popular in the sense that is very democratic. In some way it's a democratization of something that can only remain within a very specific kind of working environment*.

As with Patrizio, a large number of participants referred to word processors as essential tools for writing (Extract 3). The specific tool used to write could depend on the situation, mainly when writing collaboratively. Scholars' collaborative research practices are understudied, complex spaces (Given and Willson, 2015). In this extract, Patrizio's experience with Google Docs and Dropbox highlight that technology tools can provide new affordances to scholars to enable collaboration, providing in-built features for communication and sharing. The affordance of these two different digital objects is very similar, sharing and working online with documents, but the emotional attachment is different. In the first one, Google drive allows collaborative and simultaneous work with many researchers on the same materials, although this causes several problems for Patrizio. This instant document can generate not positive emotional attachment between him, the digital object, and the rest of the socio-material network.

**Figure 8:**
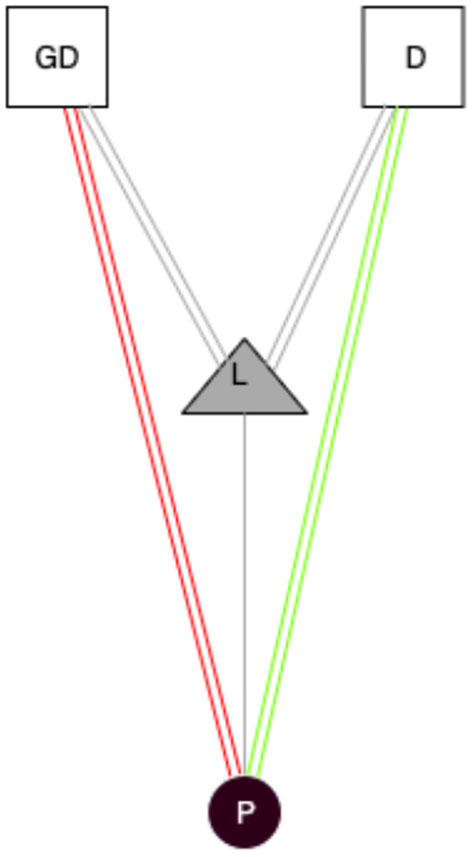
Emotional attachment of socio-material network of Patrizio. Circle, actors (P, Patrizio); Triangle, analog objects (C, Computer); Squares, digital objects (GD, Google Docs; D, Dropbox); Gray lines, usage of analog objects; Red double lines, not positive emotional attachment of usage of digital objects; Green double lines, positive emotional attachment of usage of digital objects; Gray double lines, analog/digital links.

On the other hand, we have Dropbox. In Dropbox, a researcher works on a document that can be left in the depository, and they have the history of this document. They are sure to not lose important information, Patrizio says. The emotional attachment in the socio-material network can foresee the use that tool gives to the actors. We have collected several interviews that confirm it and, as we have explained before, homophily is an essential aspect. Objects' qualities and features can inhibit or stimulate collaborations with other researchers. A tool like Dropbox, for Patrizio, can expand his scientific network and create a huge usage degree with the object, like in Basov's network (Figure 8).

In this long extract, we have observed how all these digital objects are connected to one analog object: a computer. Of course, the area of knowledge determines the number of analog objects needed. For this, we will compare Patrizio's experience with the experience of a computational scientist, Jorge.

*Extract 4*

*Jorge: In the area of mathematics, the dean will buy a hardware environment that is quite expensive, with the FPGA, which is a configurable hardware environment where you can schedule what hardware works. The idea is to implement neural networks that are highly parallel, and one works in parallel with the others*.

*Extract 5*

*Axel: If we need to calculate something complicated with the laptop, we make a remote connection to a computer center in Europe and place the calculations in parallel*.

*Extract 6*

*Luciano: If you take away my laptop or remote control, collaborations, licenses, I will only teach physics*.

The area of knowledge of these three researchers, Computational Science for Jorge (Extract 4), Physics for Axel (Extract 5), and Luciano (Extract 6), are entirely different to the area of expertise of Patrizio, Sociology. Still, the link between the actor and the analog objects is the same. It is the only gray line in their socio-material networks. What is entirely different in these networks is the connection of the analog object to the digital objects. The affordance of a computer changes depending on the area of research. For a computational scientist, an expert in neuronal networks, this analog object connects in a parallel system, using Jorge's words, which is a complete computing environment. A similar dynamic for the physicists connects to other centers in parallel.

The emotional attachment that these three researchers perceive in comparison with Patrizio's emotional attachment is different. We understand this emotional attachment is not directly related to the link between the actor and the analog object, but produced from the triad of the relations: Actor -> Analog Object -> Digital Object. This socio-material network produces the emotional attachment where we can differentiate positive attitudes (Jorge, Axel, and Luis), or the not positive attitude (Patrizio).

The analog object is not essential for the statement of the attitude, and the most important is the digital objects that the analog object connects. The affordance, in this case, is not derived directly by the analog object that gives different possibilities to the actors. Still, it is the affordance that the analog objects offer to the access of several different objects. The connection between different types of objects instead of the connection between actors and objects.

## Conclusion

Thanks to the analysis, we have answered our two research questions. As the ethnographic information also shows, the effort of the socio-material structuring principle is practically imperceptible, hiding in the shades of apparently minor ordinary activities and links between analog and digital items, in which colleagues embed through an emotional attachment, without reflecting on it. Physical engagement manages to be spread more or less similarly among the researchers. Furthermore, the sharing of items is affected by similarity in characteristics that lead to the likeness of material choices.

We have observed how researchers use technology in different areas of knowledge, but with varying levels of expertise. Speech, socio-material networks, and affordance of objects, first mostly analog and currently digital, constitute background practices for researchers. The use of objects and its affordance to the researchers has been observed in context, as an element of the socio-material networks. Overall, researchers are not satisfied with stand-alone, single-purpose items; they need objects that can be combined, working together in an interconnected setting (Given and Willson, 2018). Also, although cooperation is possible without digital technology, collaboration can be assisted through the affordances made accessible by digital instruments. The development of new tools to improve traditional work, and support new ways of working, looks to be an emergent discipline for future study in different areas. Our work contributes to the understanding of collaboration within research groups, although, in addition, it would be possible to inquire about the frequent cooperation between professionals beyond objects and their affordance. The final purpose of this research is to provide useful ways to promote management and relational strategies in different professional fields, and not only in the scientific one.

We offered a qualitative method socio-material network study to observe the interaction between analog and digital objects and researchers. The affordance is key to understand how this materiality affects the practice of the researcher. We have observed that most times it is relevant only for the digital objects, and not for the analog object. This can be studied in the future, to understand how digital objects create a stronger emotional attachment in respect to the analog. Adapting objects and digital settings to suit certain tasks is important for researchers who work frequently with technologies as a combined part of their work.

Our research has several limitations. First of all, ethnographic data on links usage is inexorably culturally arbitrated because of the presence of the viewer and the recording activities involved. Further investigation is needed to reflect ways to take into account the consequence of culture on the interaction between social links and materiality. Furthermore, the strength of a mixed ethnographic dataset lies in the containing of not only described material practices, but also the ones observed by arena scientists (Basov, 2018). Often, usages of analog and digital tools are not recognized. Also, we have not conducted a statistical survey network like in Basov's study (Basov, 2018), but have only compared the areas of similar research to define which object and which emotional attachments are defined by the link between actor and object. In the future, we must follow his longitudinal study studying the twin interaction between the physical and the social. Although here we only accounted for the effect of social structure on the physical structure, there might also be an opposite effect of shared practices and objects (Callon et al., 1986; Latour, 2005; Basov, 2018). While this study observed a variety of researchers' practices of analog and digital objects in research practice, the research is limited in its reliance on a small group of researchers mostly in Latin America and Southern European countries. The debates of objects and practices used often in academic practice were insightful. Observation of academic labor would have afforded a more complete image of the several habits researchers use in the different areas of research.

## Data Availability Statement

The raw data supporting the conclusions of this article will be made available by the authors, without undue reservation.

## Ethics Statement

The studies involving human participants were reviewed and approved by Juergen Reichardt, Yachay Tech University. The patients/participants provided their written informed consent to participate in this study. Written informed consent was obtained from the individual(s) for the publication of any potentially identifiable images or data included in this article.

## Author Contributions

The author confirms being the sole contributor of this work and has approved it for publication.

## Conflict of Interest

The author declares that the research was conducted in the absence of any commercial or financial relationships that could be construed as a potential conflict of interest.

## Publisher's Note

All claims expressed in this article are solely those of the authors and do not necessarily represent those of their affiliated organizations, or those of the publisher, the editors and the reviewers. Any product that may be evaluated in this article, or claim that may be made by its manufacturer, is not guaranteed or endorsed by the publisher.
